# A Novel PARP Inhibitor L-2286 in a Rat Model of Impact Acceleration Head Injury: An Immunohistochemical and Behavioral Study

**DOI:** 10.3390/ijms11041253

**Published:** 2010-03-26

**Authors:** Erzsébet Kövesdi, Péter Bukovics, Valérie Besson, József Nyirádi, János Lückl, József Pál, Balázs Sümegi, Tamás Dóczi, István Hernádi, András Büki

**Affiliations:** 1 Department of Neurosurgery, Medical Faculty, University of Pécs, 7623 Pécs, Hungary; E-Mails: Erzsebert.Kovesdi.CTR@usuhs.mil (E.K.); sator77@gmail.com (P.B.); jozsef.nyiradi@gmail.com (J.N.); janluc06@gmail.com (J.L.); jozsef.pal@aok.pte.hu (J.P.); tamas.doczi@aok.pte.hu (T.D.); 2 Laboratoire de Pharmacologie de la Circulation Cérébrale, UPRES EA 2510, Université René Descartes, Paris, France; E-Mail: valerie.besson@univ-paris5.fr (V.B.); 3 Department of BioChemistry, University of Pécs, 7624 Pécs, Hungary; E-Mail: balazs.sumegi@aok.pte.hu (B.S.); 4 Department of Experimental Zoology and Neurobiology, University of Pécs, 7624, Hungary; E-Mail: hernadi@ttk.pte.hu (I.H.)

**Keywords:** PARP-inhibitor, impact acceleration model, traumatic brain injury

## Abstract

We examined the neuro/axono-protective potential of a novel poly (ADP-ribose) polymerase (PARP) inhibitor L-2286 in a rat impact acceleration brain injury model. Male Wistar rats (n = 70) weighing 300–350 grams were used to determine the most effective intracerebroventricular (i.c.v.) dose of L-2286 administered 30 min after injury, and to test the neuroprotective effect at two time points (immediately, and 30 min after injury). The neuroprotective effect of L-2286 was tested using immunohistochemical (amyloid precursor protein and mid-sized mouse anti-neurofilament clone RMO-14.9 antibody) and behavioral tests (beam-balance, open-field and elevated plus maze). At both time-points, a 100 μg/rat dose of i.c.v. L-2286 significantly (p < 0.05) reduced the density of damaged axons in the corticospinal tract and medial longitudinal fascicle compared to controls. In the behavioral tests, treatment 30 min post-injury improved motor function, while the level of anxiety was reduced in both treatment protocols.

## 1. Introduction

Poly (ADP-ribose) polymerase (PARP) plays a central role in the caspase-independent apoptosis pathway mediated by apoptosis-inducing factor (AIF), an evolutionarily conserved mitochondrial flavoprotein that can be released from mitochondria after mitochondrial membrane depolarization [1]. AIF-mediated apoptosis occurs in neurons under conditions of oxidative stress [2], in experimental traumatic brain injury (TBI) [3] and brain ischemia [4]. PARP inhibition represents a therapeutic strategy targeting both necrosis, if administered early enough, and apoptosis after TBI [5]. PARP-inhibitors, such as benzamide analogs and isoquinoline derivatives, have been used previously to investigate the role of this nuclear enzyme in traumatic brain injury [5–8]. Systemic administration of the PARP-inhibitor INH_2_BP improved functional outcome after TBI in mice, but higher doses further inhibiting PARP activity worsened memory performance [5]. The PARP-inhibitor, GPI 6150, was also shown to have a protective effect in an experimental model of TBI. Besson and coworkers [8] demonstrated that PARP-1 inhibition with the PARP-inhibitors phenanthridinone-based PJ34 and isoquinolinone-based INO-1001 efficiently inhibits TBI associated with lateral fluid percussion in rats.

**Figure 1.**
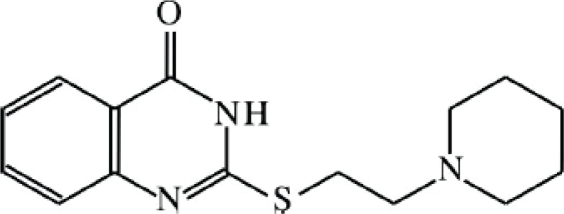
Chemical structure of L-2286 [9].

The recently developed quinazolinone derivative L-2286 (2-[(2-piperidin-1-ylethyl)thio]quinazolin-4(3*H*)-one) is considered to be a potent inhibitor of ischemia-reperfusion-induced myocardial injury [9], (Figure 1.). The aim of the present study was to determine the dose-response curve for intracerebroventricular (i.c.v.) L-2286 in an impact-acceleration (IA) model of TBI, and then to develop an efficient post-injury administration paradigm by applying the most effective dose.

## 2. Results and Discussion

### 2.1. Dose-Response Curve of i.c.v. L-2286 after Impact Acceleration Injury

**Figure 2.**
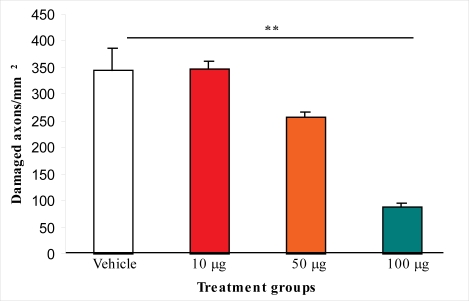
The effect of i.c.v. L-2286 administered in different doses 30 min after severe impact acceleration head injury on the density of APP immunopositive axons in the CSpT (** p < 0.01 compared to vehicle group).

In the first experiment, we found that 10 μg/rat of i.c.v. L-2286 was ineffective for reducing the density of amyloid precursor protein (APP) immunoreactive (IR) axons in the corticospinal tract (CSpT). Although 50 μg/rat of i.c.v. L-2286 reduced the density of damaged profiles, a significant difference could not be demonstrated. Ultimately, 100 μg/rat of i.c.v. L-2286 significantly reduced the APP-IR axons in CSpT (Figure 2).

### 2.2. Results of Immunohistochemical Analysis

The immunohistochemical examination, and the following digital data assessment and statistical analysis, revealed that 100 μg/rat of i.c.v. L-2286 in both treatment paradigms significantly reduced the density of APP and mid-sized mouse anti-neurofilament clone RMO-14.9 (RMO-14) IR axons after head injury.

**Figure 3.**
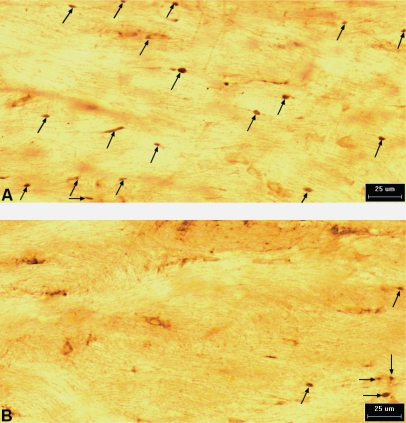
APP-IR axonal profiles (arrows) from CspT two hours post-injury. Note that the density of damaged axons appears reduced in rats treated with 100 μg/rat of i.c.v. L-2286 (B) compared to those treated with vehicle (A).

**Figure 4.**
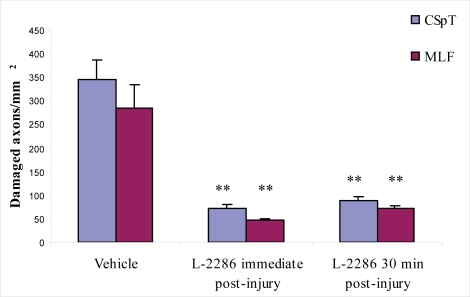
Densities of APP-immunopositive axons in CSpT and MLF in animals treated with 5 μl/rat of i.c.v. vehicle and 100 μg/rat of i.c.v. L-2286 immediately and 30 min post-injury (**p < 0.01 compared to vehicle).

**Figure 5.**
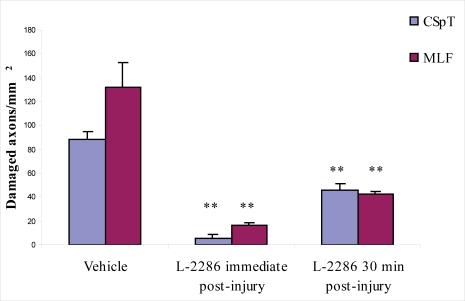
Densities of RMO-14 immunopositive axons in CSpT and MLF in animals treated with 5 μl/rat of i.c.v. vehicle and 100 μg/rat of i.c.v. L-2286 immediately and 30 min post-injury (**p < 0.01 compared to vehicle).

Treatment immediately and 30 min post-injury reduced the density of APP-IR axons (damaged axons/mm^2^) in both the CSpT and medial longitudinal fascicle (MLF) to a similar degree compared to vehicle-treated animals. Both treatment time points were effective in terms of significantly reducing the density of RMO-14-IR axons in CSpT and MLF compared to vehicle-treated animals. However, immediate post-injury treatment seems to be more effective, since a significant difference was observed in the density of damaged axons in both CSpT and MLF compared to 30 min post injury (Figure 3–5).

### 2.3. Effect of 100 μg/rat of i.c.v. L-2286 on Beam-Balance Test Performance after IA

**Figure 6.**
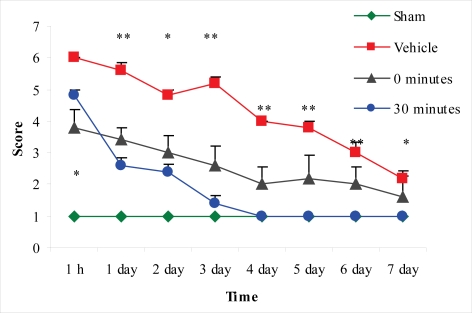
Effect of 100 μg/rat of i.c.v. L-2286 on beam-balance test scores from one hour to seven days after severe IA head injury (*p < 0.05 and **p < 0.01 compared to vehicle-treated animals).

In the assessment of the beam-balance test, sham-injured animals did not display any motor impairment. The vehicle-treated group had the worst motor performance of the four groups assessed. Both treatment paradigms improved the beam-balance performance. Immediate post-injury treatment significantly improved the motor performance at one hour. However, a statistically significant difference was not observed after this time-point in comparison to vehicle-treated rats; rats in this treatment group did not reach the maximal accessible score (1) during the seven-day period. The group treated 30 min post-injury reached the maximal accessible score on the fourth day. The motor performance of this group was significantly improved from the second examined time-point (one hour) up to the last time-point (seventh day) compared to the vehicle-treated group (Figure 6).

### 2.4. Effect of 100 μg/rat of i.c.v. L-2286 on Open-Field Performance after IA

**Figure 7.**
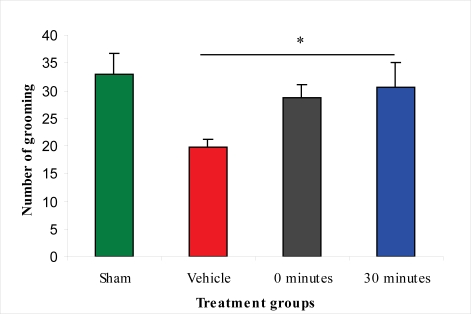
Effect of 100 μg/rat of i.c.v. L-2286 on grooming in open-field test (*p < 0.05 compared to vehicle-treated animals).

The open-field test measures altered motor performance and the level of anxiety. Results of the test showed that there were significantly fewer crossing and grooming activities in the vehicle-treated group than in the sham-injured group. Immediate post-injury treatment with L-2286 did not significantly improve any of the three examined parameters compared to vehicle-treated animals. However, L-2286 treatment 30 min post-injury significantly improved the number of grooming activities compared to the vehicle-treated group, but without any effect on the another two parameters (crossing and rearing) (Figure 7).

### 2.5. Effect of 100 μg/rat of i.c.v. L-2286 on Elevated Plus-Maze Performance after IA

**Figure 8.**
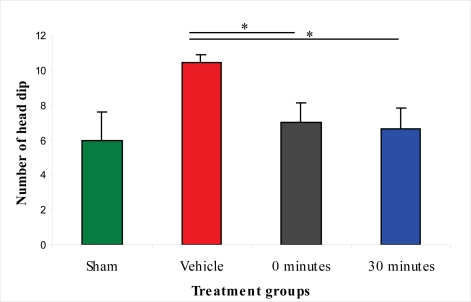
Effect of 100 μg/rat of i.c.v. L-2286 on head dip in the elevated plus-maze test (*p < 0.05 compared to vehicle-treated animals).

**Figure 9.**
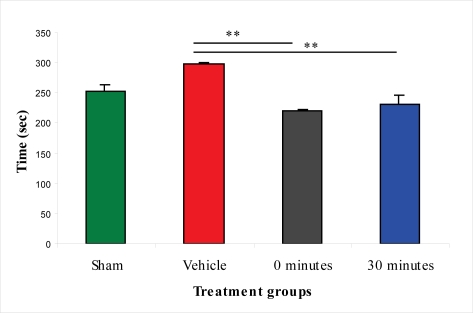
Effect of 100 μg/rat of i.c.v. L-2286 on time spent in the closed arm in the elevated plus-maze test (**p < 0.01 compared to vehicle-treated animals).

**Figure 10.**
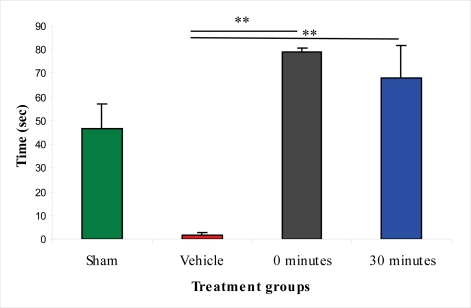
Effect of 100 μg/rat of i.c.v. L-2286 on time spent in the open arm in the elevated plus-maze test (**p < 0.01 compared to vehicle-treated animals).

Results of the elevated plus-maze test indicate that the vehicle-treated animals suffered from anxiety after injury. A statistically significant difference between the sham-injured and vehicle-treated groups was detected in the number of head dips in the open arm, time in the open arm, time in the closed arm, and the amount of grooming in the closed arm. The anxiety level was significantly lower in the L-2286-treated group than in the vehicle treated animals, since rats spent significantly more time in the “unsafe” open arm than in the “safe” closed arm. Interestingly, the group treated immediately post-injury not only spent significantly more time in the open arm than the vehicle treated group, but also the sham-injured group, a finding not observed in rats treated 30 min post-injury. The vehicle-treated and the latter group significantly differed only in the parameters of head dips in the open arm, time in the open arm and time in the closed arm (Figure 8–10).

### 2.6. Discussion

In our study, we demonstrated that the novel PARP inhibitor L-2286 is capable of preventing both morphological (axonal) and neurobehavioral sequelae associated with severe TBI evoked in a rat IA head injury model.

The first stage of the study determined the effective dose of this new drug in a post-injury administration paradigm using immunohistochemical analysis of APP accumulation in CSpT. This assay was chosen as APP is recognized as the primary (classical) marker of diffuse axonal injury (DAI) evoked by TBI. Intracerebroventricular treatment with different doses of L-2286 30 min post-injury proved that 100 μg/rat is the most effective dose of those applied (10; 50; 100 μg).

In the second phase of the experiment, APP and RMO-14 immunohistochemistry was used to further elucidate the efficiency of 100 μg/rat of i.c.v. L-2286 administered at time points after TBI (immediately and 30 min post-injury). Both time points were effective in terms of significantly reducing the densities of damaged axons in CSpT and MLF. It should be noted, however, that in the case of RMO-14-IR axons, immediate post-injury treatment appeared to be far more effective, although this difference was not observed in APP-IR axons.

To the best of our knowledge, this study is the first attempt to assess the efficacy of PARP-inhibition on DAI. Studies have thus far aimed at analyzing poly(ADP-ribose) immunohistochemistry [8] or changes in the volume of brain lesions [8,10] following TBI. Since traumatic axonal injury (TAI) has two major morphological characteristics assessable by immunochemistry, *i.e*., the formation of axonal bulbs (predominantly detected by APP-IR) and cytoskeletal alterations reflected in neurofilament compaction (RMO-14-IR) [11,12], which may also occur independently in subpopulations of injured axons, we applied both immunohistochemical strategies to detect diffuse axonal injury in a reliable fashion.

Our qualitative and quantitative immunohistochemical results corroborate earlier observations that show the complexity of TAI pathogenesis. Specifically, we demonstrated that RMO-14 and APP-IR axon populations display different reactions to the same neuroprotective intervention, a finding well explained in the aforementioned studies.

Based on the finding that there is a strong decrease in the density of damaged IR axonal profiles, we assessed the functional efficacy of the therapeutic strategies applied. Many articles have demonstrated that Marmarou’s impact acceleration model impairs cognitive and motor functions in rodents [13–15]. The neurobehavioral outcome after TBI has primarily been examined with beam-walking, beam-balance, inclined-plane, Morris water-maze and Barnes-maze tests [16–18]. In our study, motor-behavioral tests after administration of 100 μg/rat of i.c.v. L-2286 significantly improved motor function and decreased anxiety levels. Specifically, 100 μg/rat of i.c.v. L-2286 in the beam-balance test for motor performance after TBI shows that 100 μg/rat of i.c.v. L-2286 administered 30 min after injury is even more effective than the immediate post-injury treatment, a finding that requires further investigation. In the open-field test assessing motor performance and anxiety-levels, the crossing and grooming activity of the vehicle-treated group was significantly lower than that of the sham-injured animals. A dose of 100 μg/rat of i.c.v. L-2286 administered 30 min post-injury significantly improved the amount of grooming, while the six other monitored parameters were not affected. In the elevated plus-maze test, vehicle-treated animals displayed anxiety after the injury, spending less time in the “unsafe” open arm. Vehicle-treated rats showed a clear preference for the “safe” closed arm during this test compared to the sham-injured and drug-treated groups. Nevertheless they still displayed some curiosity as the number of head dips into open arm is significantly higher than that of the other groups. Rats that have been treated with 100 μg/rat of i.c.v. L-2286 immediately post-injury spent significantly more time in the open (“less safe”) arm than their controls, suggesting that this relative risk-taking behavior might be associated with a side-effect of the drug. This has been postulated by Satchell and coworkers [5], who demonstrated in their experiment that high-dose PARP-inhibitors worsened memory performance. Interestingly enough, this “side effect” disappeared in the group treated 30 min post-injury.

Application of PARP-inhibitors may be useful in the treatment of numerous neurodegenerative diseases. The brain is particularly susceptible to radical-mediated neuronal damage due to high levels of oxygen consumption, unsaturated fatty acids, and iron stores, combined with low antioxidant resources. Oxidative stress is a critical step in neuronal degeneration [19–21]. Iwashita and coworkers [21] studied a newly synthesized PARP-inhibitor, FR247304, and found that this drug could exhibit potent PARP-1 inhibition both *in vitro* and *in vivo*, with significant neuroprotective properties following ischemia-reperfusion in rats. This suggests that this compound or its derivative could not only be an important tool to investigate the physiological role of PARP in neurodegenerative pathways, but also a therapeutic option for stroke and neurodegenerative disease.

Provided that PARP activation and nicotinamide-adenine-dinucleutide (NAD^+^) depletion contribute to cell death, its absence or inhibition may prevent cell death associated with the apoptotic cascade and energy depletion. There is solid evidence for the beneficial effects of energy conservation (*via* NAD^+^ conservation) with PARP-inhibition in ischemic insults to the brain [22–26], but this theory requires further investigation in TBI. PARP inhibition following TBI may be neuroprotective in the acute period (<2 h post-injury in the rodent), when PARP activity is elevated. Chronic inhibition of PARP as a neuroprotective strategy results in behavioral recovery, although it may differentially affect brain subregions.

PARP-inhibitors have been shown to improve neurological outcome in models of head injury [5,6,8,10]. Satchell and coworkers [5] administered intraperitoneal (i.p.) INH_2_BP immediately after injury and one day after controlled cortical impact (CCI) brain injury in mice. They found improved Morris water-maze performance with no difference in contusion volume, hippocampal neuron survival, or motor performance. Examining the effect of i.p. INO-1001 30 min before and three times daily for three days after fluid percussion injury in rats, Besson and coworkers [8] found improved motor function. In Clark’s [10] experiments, INO-1001 reduced the latency in mice to find the hidden platform in Morris water-maze and increased the time spent in the target quadrant after CCI. Ding and coworkers [27] demonstrated that 3-aminobenzamide (3-AB), a selective inhibitor of PARP, significantly reduced brain damage after focal ischemia in rats and also improved impaired motor functions. PARP-inhibitor 1,5-isoquinolinediol, treatment alleviates, but does not completely normalize, tail-flick and paw-withdrawal response latencies, mechanical and tactile withdrawal thresholds, and exaggerated flinching behavior in rats with short-term streptozotocin-induced diabetes [28]. In hypoglycemia, significant learning and memory deficits were identified with behavioral testing six weeks after injury. Animals treated with 3-AB after hypoglycemia did not show any significant deficit in their ability to locate the platform compared with sham-operated animals during either the visible or hidden platform trials. The behavioral and histological studies after hypoglycemia suggest that the neuroprotection provided by PARP inhibition leads to long-lasting preservation of neuronal survival and function [29].

As maintenance of energy homeostasis in the prevention of DAI has been proven to be of ample importance [30–33], it seemed logical to assess the efficacy of this novel PARP inhibitor (L-2286) in the widely used model of impact acceleration (diffuse) brain injury described by Marmarou.

## 3. Experimental Section

Sixty-eight adult male Wistar rats weighing 300–350 g (Charles River, Budapest, Hungary) were used for the experiments. Animals were housed in single cages under controlled environmental conditions (22 °C, 12-12 h light-dark cycle) with food and water *ad libitum* for two weeks prior to the study. The experiments were carried out in accordance with regulations of the Hungarian Animal Care Committee (BA02/2000-26/2001).

### 3.1. Injury Induction

The injury protocol was the same in all experiments. All rats were first anesthetized in a bell jar for 5 min with 4% isoflurane (Forane, Abott, Hungary) in 70% N_2_O and 30% O_2_. After endotracheal intubation, rats were ventilated with 1.5% isoflurane in 70% N_2_O and 30% O_2_ (Inspira ASV, Harvard Apparatus USA). Arterial oxygen saturation was measured *via* pulse oximetry (Nonin 8600V), rectal and temporal temperatures were monitored and maintained at 37 °C with a feedback-controlled heating pad (FHC BOWDOINHAM ME 04008 USA Temperature Control). A midline incision was made to expose the skull from the bregma to the lambda sutures. A stainless steel disc (10 mm in diameter and 3 mm thickness) was fixed centrally between the lambda and bregma sutures using cyanoacrylate. A 450-g weight was dropped from 2 m onto the stainless disc fixed to the rat’s skull. After impact, the metal disc was removed and the animals were monitored during the recovery of spontaneous respiration. Sham animals were prepared for injury in the same fashion, but were not injured.

### 3.2. Establishment of Dose-Response Curve for i.c.v. L-2286

To establish the dose-response curve for L-2286, each rat (n = 5 in each group) was given a single bolus of 10, 50 or 100 μg/rat of L-2286 dissolved in 5 μL of physiological saline. The drug was administered i.c.v. 30 min after severe head injury through a craniotomy 1 mm posterior, 1.5 mm lateral and 3.5 mm deep relative to bregma. The vehicle group (n = 5) received i.c.v. 5 μL/rat of physiological saline. Two hours post-injury, animals were sacrificed for immunohistological assessment to determine the dose-response curve in terms of a reduction in the density of damaged IR axons in the corticospinal tract.

### 3.3. Experimental Protocol to Test the Post-Injury Administration Regime

Rats (n = 24) received a single 100 μg/rat bolus of i.c.v. L-2286 dissolved in 5 μL of physiological saline, while vehicle-treated animals were treated with i.c.v. 5 μL/rat of physiological saline. L-2286 was administered either 0 min (n = 12) or 30 min (n = 12) after injury. Animals for histological analysis (n = 24) were sacrificed 2 h after brain injury. The remaining 30 animals survived 9 days post-injury and underwent behavioral tests each day.

### 3.4. Immunohistochemistry

Two hours after injury, rats were sacrificed with i.p. sodium pentobarbital and transcardially perfused with 4% paraformaldehyde in Millonig’s buffer. Brains were removed from the skull and immersed in the same fixative overnight (16–18 h). Five mm-wide blocks of the brain were cut out using a sagittal brain-blocking device (Braintree Scientific Inc.), which include the regions extending from the interpeduncular fossa to the first cervical segment and were immersed in the same fixative for 24 h. Blocks were sectioned with a Vibratome Series 1500 (Neurobionic, Denmark) at a thickness of 30 μm and collected in 0.1 M phosphate buffer in a semi-serial fashion. Adjacent sections were then processed for immunohistochemical detection of damaged axonal profiles. Impaired axoplasmatic transport, long associated with TAI, was assessed by immunohistochemical detection of APP [11,33–38]. APP is carried by fast axoplasmic transport and accumulates at the foci of disturbed axoplasmic transport. A polyclonal antiserum targeting the C-terminus of beta-APP was used for this purpose [37,38]. Sections adjacent to those single labeled for APP, were single labeled for the detection of neurofilament (NF) compaction, another marker of DAI [11,39–43]. To this end, RMO-14 antibody was used, which is known to target an epitope on the rod domain of altered NF-M subunits exposed upon modification of the NF sidearms, an assumed consequence of calcium-induced enzymatic processes [39,41,43]. After blocking endogenous peroxidase activity and rinsing in phosphate buffered saline (PBS), the above-defined groups of sections were microwaved (300 W) in citrate buffer for antigen retrieval as previously described [44]. Sections were then rinsed in PBS containing 1% normal goat serum (NGS, for APP labeling (Zymed)) or 1% normal horse serum (NHS, for RMO-14 labeling (Zymed)) and were incubated for 35 min with 0.2% Triton X (Sigma Aldrich, Hungary) in 10% NGS or NHS in PBS, respectively (Sigma Chemical Co., St Louis, MO). After two quick rinses in PBS containing 1% NGS (or NHS in the case of RMO-14), sections were incubated overnight in rabbit anti-APP antibody (1:500) (Zymed Laboratories, Hartford, CT) or in mouse monoclonal RMO-14 antibody (1:500) (Zymed Laboratories, Hartford, CT). On the following day after rinsing in PBS containing 1% NGS (or NHS), sections were incubated in biotinylated anti-rabbit immunoglobulin derived from goat (diluted 1:200 in 1% NGS/PBS) or in 1:400 dilution of biotinylated rat adsorbed anti-mouse immunoglobulin derived from horse for 60 min, followed by rinsing in PBS. After incubation in an avidin-biotin-peroxidase complex (ABC standard “Elite” kit, Vector, England, at a dilution of 1:100) and rinsing 3 × 10 min in PBS and 2 × 10 min in 0.1 M phosphate buffer, sections were processed for visualization of the immunohistochemical complex using 0.05% diaminobenzidine (DAB) and 0.01% hydrogen peroxide in 0.1 M phosphate buffer. The sections were subsequently mounted and cleared for routine light microscopic examination.

### 3.5. Image Analysis

APP or RMO-14 IR axonal profiles were examined using a NIKON light microscope interfaced with a SPOT RT digital camera and a computer-assisted image analysis system (IMAGE-PRO PLUS v5.0.1) in a blinded fashion. The area of CSpT and MLF was analyzed. Six sections containing both CSpT and MLF regions from each animal were captured and digitized at a magnification of 10×. A 180,000 μm^2^ grid was superimposed over the CSpT and adjacent MLF region, and all damaged axonal profiles within the grid were counted and expressed as density of APP or RMO-14-IR axons (IR-axons/mm^2^).

### 3.6. Behavioral Tests

Behavioral tests were conducted one day before (beam-balance) and from 1 h to 9 days after injury. The trials were scored blind by a single observer. The behavioral tests were done during the light phase. Between each trial, all apparatus were cleaned with a 75% ethanol solution.

#### 3.6.1. Beam-Balance Test

Before injury, animals were given repeated training until capable of balancing on the beam for three consecutive trials of 60 sec each. Rats were placed at the center of a 1.5-cm-wide square wooden bridge that was suspended 60 cm above a foam pillow. All animal groups were tested after the injury (one trial/rat) for 7 days (1 h and 1–7 days after injury) and scored from 1–6 on beam balance [45]. All trial scores from each group were averaged daily for statistical analysis. The scoring was as follows: 1: balances with steady posture; 2: grasps the side of the beam and/or has unsteady movements; 3: hugs the beam or slips without falling; 4: attempts to balance on the beam but falls off; 5: drops over the beam or hangs on the beam and falls off and 6: does not attempt to balance on the beam.

#### 3.6.2. Open-Field Test

The open-field test was used to measure spontaneous locomotor activity. The open-field is a box with a 70 × 70 cm floor and a height of 50 cm. On the eighth day post–injury, rats were brought into the experimental room. They remained in their transfer cages for 30 min, after which each subject was placed into the center of the open-field box. Crossing, rearing and grooming were recorded with a video camera during the 5-minute test.

#### 3.6.3. Elevated Plus-Maze Test

The elevated plus-maze was used to measure anxiogenic behavior. The maze consisted of 4 intersecting arms and was constructed from varnished wood. The maze was elevated 1 m above the floor. The arms of the maze were 45 cm long and 10 cm wide; the closed arms had walls on 3 sides that were 30 cm high, and the open arms did not have walls on any side. Nine days after the trauma, rats were placed one-by-one in the center of the maze facing one of the closed arms. An observer, who was seated 1.5 m from the apparatus, recorded with a video camera the number of head dips into the open arms, entries with the first limbs into the open arms, grooming in both the closed and open arms and the time in closed or open arms.

#### 3.6.4. Statistical Analysis

ANOVA with Dunnett’s post hoc test was used to compare the density of immunopositive axonal-profiles (expressed as mean number/mm^2^) between L-2286- and vehicle-treated animals. For beam-balance, the scores of each group were totaled and averaged daily. ANOVA with Dunn’s post hoc tests were used to compare the scores of experimental groups. For open-field, the number of crossings, rearings and the time spent in central and peripheral zones during the 5-minute test were analyzed. For elevated plus-maze, the number of head dips into the open arms, entries with the first limbs into the open arms, grooming in both closed and open arms and the time spent in closed or open arms were analyzed. Results of open-field and elevated plus-maze were analyzed with ANOVA and Dunnett’s post hoc tests. Differences were considered significant with a p value of 0.05. Statistical analysis was performed using Graph Pad Instat software.

## 4. Conclusions

Our results strongly suggest the neuroprotective effect of PARP inhibition; the heterogeneity of the results mandates further, detailed experiments that should shed light on the exact subcellular pathomechanism of this PARP inhibition. Specifically, we should elucidate the role of diffuse neural injury not assessed in the present experiments that may lay behind the observed motor-behavioral benefit provided by L-2286. To this end, further studies in different models, primarily in fluid percussion head injury, are planned, while also assessing ultrastructural details, particularly mitochondrial integrity in the axonal bundles that are prone to diffuse TBI.
